# Supplementary material for: Heparin-based hydrogel scaffolding alters the transcriptomic profile and increases the chemoresistance of MDA-MB-231 triple-negative breast cancer cells
Source: Biomater Sci. 2020 Feb 13;8(10):2786–96. doi: 10.1039/c9bm01481k (PMC7497406; doi:10.1039/c9bm01481k)
Supplement: Supplementary file 2 [file BM-008-C9BM01481K-s002.zip › Supplementary File 4/HP-BhydrogelvControl/Pathways/my_analysis.Gsea.1545201079907/HALLMARK_WNT_BETA_CATENIN_SIGNALING.html]

Details for gene set HALLMARK\_WNT\_BETA\_CATENIN\_SIGNALING[GSEA]

|  || Dataset | expr.class.cls#HYD\_versus\_CONTROL.class.cls#HYD\_versus\_CONTROL\_repos |
| Phenotype | class.cls#HYD\_versus\_CONTROL\_repos |
| Upregulated in class | HYD |
| GeneSet | HALLMARK\_WNT\_BETA\_CATENIN\_SIGNALING |
| Enrichment Score (ES) | 0.47037634 |
| Normalized Enrichment Score (NES) | 1.5064656 |
| Nominal p-value | 0.02255639 |
| FDR q-value | 0.068143085 |
| FWER p-Value | 0.295 |
Table: GSEA Results Summary

  

Fig 1: Enrichment plot: HALLMARK\_WNT\_BETA\_CATENIN\_SIGNALING      
 Profile of the Running ES Score & Positions of GeneSet Members on the Rank Ordered List

  

| PROBE | DESCRIPTION (from dataset) | GENE SYMBOL | GENE\_TITLE | RANK IN GENE LIST | RANK METRIC SCORE | RUNNING ES | CORE ENRICHMENT || 1 | AXIN1 | na |  |  | 639 | 2.169 | 0.0392 | Yes |
| 2 | FRAT1 | na |  |  | 681 | 2.131 | 0.1084 | Yes |
| 3 | NOTCH1 | na |  |  | 1056 | 1.835 | 0.1503 | Yes |
| 4 | JAG2 | na |  |  | 1375 | 1.640 | 0.1885 | Yes |
| 5 | LEF1 | na |  |  | 1417 | 1.622 | 0.2407 | Yes |
| 6 | MAML1 | na |  |  | 1653 | 1.509 | 0.2789 | Yes |
| 7 | DVL2 | na |  |  | 1671 | 1.502 | 0.3282 | Yes |
| 8 | NCOR2 | na |  |  | 1743 | 1.474 | 0.3738 | Yes |
| 9 | TCF7 | na |  |  | 2121 | 1.307 | 0.3979 | Yes |
| 10 | PPARD | na |  |  | 2220 | 1.265 | 0.4351 | Yes |
| 11 | NOTCH4 | na |  |  | 3162 | 0.998 | 0.4194 | Yes |
| 12 | KAT2A | na |  |  | 3384 | 0.943 | 0.4394 | Yes |
| 13 | MYC | na |  |  | 3771 | 0.854 | 0.4478 | Yes |
| 14 | PSEN2 | na |  |  | 3877 | 0.838 | 0.4704 | Yes |
| 15 | PTCH1 | na |  |  | 5058 | 0.618 | 0.4295 | No |
| 16 | WNT5B | na |  |  | 5088 | 0.615 | 0.4485 | No |
| 17 | HDAC5 | na |  |  | 5924 | 0.497 | 0.4216 | No |
| 18 | AXIN2 | na |  |  | 6092 | 0.472 | 0.4286 | No |
| 19 | CSNK1E | na |  |  | 7627 | 0.288 | 0.3582 | No |
| 20 | GNAI1 | na |  |  | 7970 | 0.251 | 0.3488 | No |
| 21 | FZD8 | na |  |  | 8335 | 0.215 | 0.3370 | No |
| 22 | FZD1 | na |  |  | 8501 | 0.206 | 0.3353 | No |
| 23 | SKP2 | na |  |  | 8588 | 0.194 | 0.3373 | No |
| 24 | CCND2 | na |  |  | 9609 | 0.088 | 0.2870 | No |
| 25 | NUMB | na |  |  | 10861 | -0.047 | 0.2233 | No |
| 26 | ADAM17 | na |  |  | 11082 | -0.075 | 0.2144 | No |
| 27 | HDAC11 | na |  |  | 11503 | -0.112 | 0.1962 | No |
| 28 | DKK1 | na |  |  | 12791 | -0.256 | 0.1376 | No |
| 29 | CUL1 | na |  |  | 14693 | -0.522 | 0.0559 | No |
| 30 | NCSTN | na |  |  | 14834 | -0.542 | 0.0667 | No |
| 31 | JAG1 | na |  |  | 15843 | -0.713 | 0.0379 | No |
| 32 | TP53 | na |  |  | 16860 | -0.944 | 0.0165 | No |
| 33 | CTNNB1 | na |  |  | 16890 | -0.952 | 0.0468 | No |
| 34 | HDAC2 | na |  |  | 16976 | -0.979 | 0.0751 | No |
| 35 | RBPJ | na |  |  | 17645 | -1.218 | 0.0810 | No |
Table: GSEA details [plain text format]

  

Fig 2: HALLMARK\_WNT\_BETA\_CATENIN\_SIGNALING      
 Blue-Pink O' Gram in the Space of the Analyzed GeneSet

  

Fig 3: HALLMARK\_WNT\_BETA\_CATENIN\_SIGNALING: Random ES distribution      
 Gene set null distribution of ES for **HALLMARK\_WNT\_BETA\_CATENIN\_SIGNALING**
